# Genome-Wide Identification and Characterization of Chinese Cabbage S1fa Transcription Factors and Their Roles in Response to Salt Stress

**DOI:** 10.3390/antiox11091782

**Published:** 2022-09-09

**Authors:** Ali Anwar, Shu Zhang, Li-Xia Wang, Fengde Wang, Lilong He, Jianwei Gao

**Affiliations:** Shandong Branch of National Vegetable Improvement Center, Institute of Vegetables, Shandong Academy of Agricultural Sciences, Jinan 250100, China

**Keywords:** S1fa transcription factor, cell wall, yeast; antioxidant enzyme, ROS, salt stress

## Abstract

The S1fa transcription factor is part of a small family involved in plant growth and development and abiotic stress tolerance. However, the roles of the S1fa genes in abiotic stress tolerance in Chinese cabbage are still unclear. In this study, four S1fa genes in the Chinese cabbage genome were identified and characterized for abiotic stress tolerance. Tissue-specific expression analysis suggested that three of these four S1fa genes were expressed in all tissues of Chinese cabbage, while *Bra006994* was only expressed in the silique. Under Hg and Cd stresses, the S1fa genes were significantly expressed but were downregulated under NaCl stresses. The *Bra034084* and *Bra029784* overexpressing yeast cells exhibited high sensitivity to NaCl stresses, which led to slower growth compared with the wild type yeast cells (EV) under 1 M NaCl stress. In addition, the growth curve of the *Bra034084* and *Bra029784* overexpressing cells shows that the optical density was reduced significantly under salt stresses. The activities of the antioxidant enzymes, SOD, POD and CAT, were decreased, and the MDA, H_2_O_2_ and O_2_^−^ contents were increased under salt stresses. The expression levels of cell wall biosynthesis genes *Ccw14p*, *Cha1p*, *Cwp2p*, *Sed1p*, *Rlm1p*, *Rom2p*, *Mkk1p*, *Hsp12p*, *Mkk2p*, *Sdp1p* and *YLR194c* were significantly enhanced, while *Bck1p*, and *Ptc1p* were downregulated under salt stresses. These results suggest that the *Bra034084* and *Bra029784* genes regulate cell wall biosynthesis and the defense regulatory system under salt stresses. These findings provide a fundamental basis for the further investigation of crop genetic modification to improve crop production and abiotic stress tolerance in Chinese cabbage.

## 1. Introduction

Chinese cabbage (*Brassica rapa*) is a winter vegetable crop that originated in China and is mainly cultivated in north China [1,2]. It is the largest vegetable crop that is produced throughout the year, and because of its excellent nutritional value, it is highly consumed [3]. As a leafy vegetable, Chinese cabbage is more sensitive to environmental influences, including NaCl, heavy metals, low and high temperature, etc., which cause a series of physiological, molecular and biochemical changes that negatively affect plant growth and production [4,5]. To cope with different environmental influences, molecular, cellular and biochemical responses are regulated through many series of pathways, inducing antioxidant enzymes, hormones and transcription factors (TFs), to reduce the detrimental effects of stresses [6]. Under abiotic stresses, the plant produces ROS (reactive oxygen species), which is highly toxic and reactive, and hence causes oxidative damage and cell death [7]. Plant cells exhibit high efficiencies in scavenging ROS through the well-established coordination of antioxidant enzymes (SOD, POD, CAT, GS, APX and GR) and non-enzymatic antioxidants (ascorbic acid and glutathione) [8]. The overproduction of ROS initially causes damaged cells, hormonal imbalance and decreased metabolic and enzymatic activities, and thus interferes with signaling pathways that are involved in carbohydrate, protein, lipid, chlorophyll and photosynthetic machinery [6,7,8].

Plants have established a wide range of physiological and biochemical mechanisms to avoid the harmful effects of environmental influences [9,10]. The overproduction of ROS is diminished through the regulation of gene and protein expression levels in the mitochondria, nucleus, chloroplast and cell wall [11]. The cell wall is a complex structure, which is the first and central protective barrier for abiotic stress [11]. A number of signaling cascades have been reported to be involved in the regulation of stress responses in cell walls, including cell wall integrity (CWI), high-osmolality glycerol (HOG) and protein kinase A (PKA) [11]. *AtMyb41* regulates osmotic and salinity stresses through the activation of cell wall biosynthesis genes [12]. Rice *R2R3-type* MYB transcription factor, *OsMPS*, negatively regulates the expression of hormone signaling genes, EXPANSIN and biosynthesis genes in the cell wall to enhance NaCl stress tolerance [13]. Wall-associated kinases (WAKs), a potential sensor for the cell wall, regulate the pectic signaling network of the cell wall under abiotic stresses [14].

Transcription factors are crucial regulators of plant abiotic stresses, and are involved in the regulation of the defense system to stabilize ROS production [9,15,16]. Among different transcription factors, the S1fa transcription factor is highly conserved and belongs to the smallest family of the plant kingdom [17]. The members in this family have a small molecular weight (7 to 9 KD) and an average length of 70 to 80 aa, and are mainly localized in the nucleus [17]. Although usually no more than five S1fa proteins are found in most plants, such as maize, rice, tomato, soybean, *Arabidopsis* and Chinese cabbage, *Arachis duranensis* has 126 S1fa proteins [18]. Chinese cabbage has four members of the S1fa proteins, including *Bra003132*, *Bra034084*, *Bra006994* and *Bra029784*, which bind to the cis-element of the site 1 binding site, one of the three highly conserved binding sites (site 1, 2 and 3) located in the promoter region. Previous studies have reported that spinach S1fa has a nuclear localization signal peptide and a DNA recognition motif, which may function as a transcription factor [19]. The S1fa genes are mainly expressed in roots and etiolated seedlings rather than leaves, indicating their involvement in growth and development [17,18]. The S1fa gene plays a key role in abiotic stress tolerance. Under abiotic stresses, the S1fa genes are the most downregulated genes in cotton [17]. Moreover, in Chinese cabbage, the S1fa gene shows significant responses to salinity stress, suggesting that it may act as the upstream gene for salt responsive genes. Two genes of the S1fa family, *PtS1Fa1* and *PtS1Fa2*, have been characterized in *Populous trichocarpa* [17]. The results show that the *PtS1Fa2* overexpression lines of *P. trichocarpa* increase in fresh weight, root length and chlorophyll accumulation under drought stresses. However, the overexpression of *PtS1Fa1* has no obvious effect on the drought stress response. These findings suggest that *PtS1Fa2* plays a key role in the activation of antioxidant enzymes such as SOD and POD to reduce the MDA, H_2_O_2_ and O_2_^−^ contents, and induces drought tolerance. The *OsS1fa* gene in rice confirms drought stress tolerance in *Arabidopsis* [18]. These results demonstrate that the *OsS1fa* gene is highly expressed in the leaf, culm and root. Drought tolerance-related genes, such as *LEA*, *GRF7*, *YODA*, *RD29A* and *CPK6*, are significantly expressed in the *OsS1fa* overexpression line under drought stresses, suggesting that *OsS1fa* plays a fundamental role in plant development and abiotic stress responses.

In this study, four members of the S1fa family were identified and characterized through the investigation of phylogeny, motif, gene structure, cis-element and miRNA in Chinese cabbage. Furthermore, the functions of the S1fa genes in response to abiotic stresses (Hg, Cd, Al, Co, Cu, mannitol (osmotic stress), salt and cold and heat stress) were investigated in yeast, which showed that two S1fa genes were highly sensitive to NaCl stress. The TPM value of the S1fa genes was measured in different plant tissues, such as the root, leaf, stem, flower, callus, silique and specific leaves of Chinese cabbage. The pRS416-GFP vector was used to test the subcellular localization of the S1fa genes under salinity stress in yeast. The significance of this study will be helpful for the understanding of the S1fa gene’s function and boost the genetic modification of Chinese cabbage, which can improve crop production and adaptation to environmental cues. It will be more interesting to explore the role of S1fa genes in the hormone signaling pathway, interaction and crosstalk to identify novel genes in Chinese cabbage. Future research on S1fa will offer the possibility of genetic engineering of crop varieties with enhanced crop production.

## 2. Results

### 2.1. Identification and Characterization of the S1fa Family Genes in Chinese Cabbage

**Figure 1:**
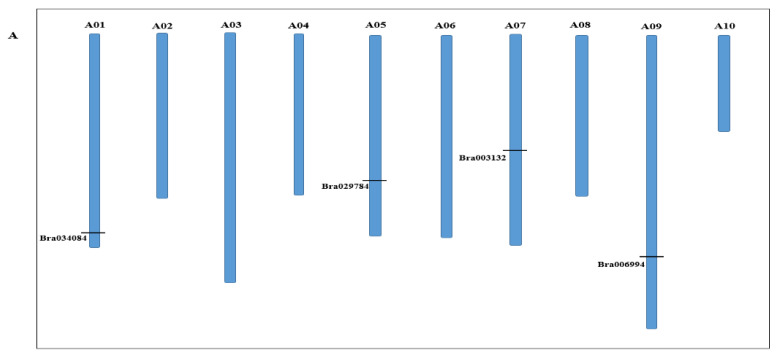

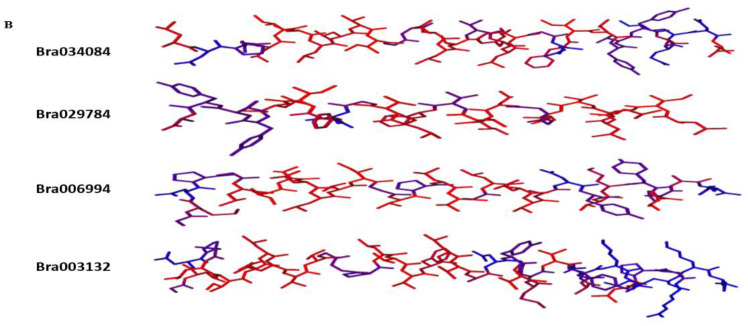
Chromosomal localization of the S1fa genes in Chinese cabbage and protein 3D structures. (**A**) Chinese cabbage has ten chromosomes, and the chromosome number (A01 to A10) is shown on the top of each chromosome. The position marked on the chromosome represents the location of the S1fa genes. (**B**) Protein 3D structure of Chinese cabbage S1fa genes.

To identify and characterize the S1fa transcription factor genes in Chinese cabbage, we performed BLASTP searches against the Chinese cabbage genome database (http://brassicadb.cn (accessed on 22 March 2022)) using three *Arabidopsis* S1fa protein sequences (AT2G37120, AT3G53370 and AT3G09735) as query sequences, and confirmed four candidate genes of the S1fa family in Chinese cabbage, including *Bra003132*, *Bra034084*, *Bra006994* and *Bra029784*. The S1fa family genes are distributed on different chromosomes of Chinese cabbage; *Bra034084, Bra029784, Bra003132* and *Bra006994* are located on A01, A05, A07 and A09, respectively (Figure 1A). The protein 3D structure of S1fa gene showed similar structural homology in Chinese cabbage (Figure 1B). The length of the S1fa genes is between 70–-88 aa, with a molecular weight ranging from 7.8 to 9.3 kDa (Table 1). The isoelectric point of Chinese cabbage S1fa proteins is relatively high (pI > 10.38), indicating that they are rich in alkaline amino acids (Table 1). Subcellular location analysis showed that all S1fa genes were localized in the nucleus.

### 2.2. Phylogenetic Analysis of the S1fa Genes in Chinese Cabbage

**Figure 2:**
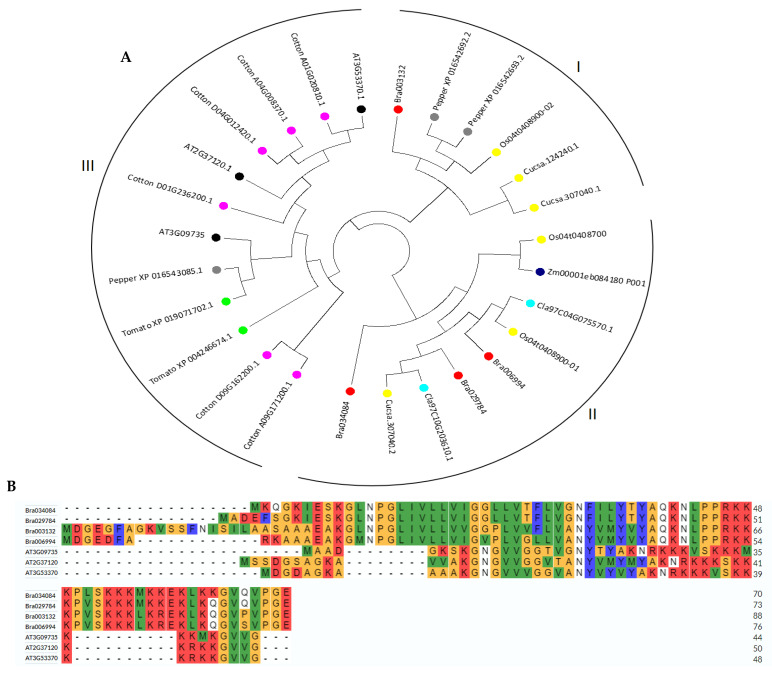
Phylogenetic analysis and multiple sequence alignment of the S1fa proteins. (**A**) Phylogenetic tree of the S1fa genes in Chinese cabbage, tomato, pepper, cotton, rice, *Arabidopsis*, cucumber, watermelon and rice. The neighbor-joining tree was generated using the MEGA7 software with 100 bootstrap replicates. The different colored dots represent different plant species. (**B**) Multiple sequence alignment of S1fa proteins from Chinese cabbage and *Arabidopsis*. Multiple sequence alignment was performed using the MEGA7 software. The highlighted amino acids are highly conserved.

The phylogenetic analysis was used to investigate the evolution of the S1fa genes in Chinese cabbage. The S1fa proteins were compared with those in other species, including tomato, pepper, cotton, rice, *Arabidopsis*, cucumber, watermelon and rice, to investigate and explore the evolutionary relationships. A total of 27 S1fa proteins were clustered into three groups (I, II and III), which consisted of 6, 9 and 12 members, respectively. *Bra003132* was clustered into group I, while *Bra034084*, *Bra006994* and *Bra029784* were clustered into group II, indicating that Chinese cabbage S1fa genes have high homology with those in rice, cucumber and pepper (Figure 2A). Additionally, low bootstrap values in the phylogenetic tree are due to the divergence of the protein sequences that occur between Chinese cabbage and *Arabidopsis*, cotton and tomato during the evaluation. Multiple sequence alignments show that the amino acid sequences of the S1fa genes are highly conserved between Chinese cabbage and *Arabidopsis* (Figure 2B). The conserved domain of S1fa is highlighted in Figure 2B.

### 2.3. Cis-Element Analysis of S1fa

**Figure 3:**
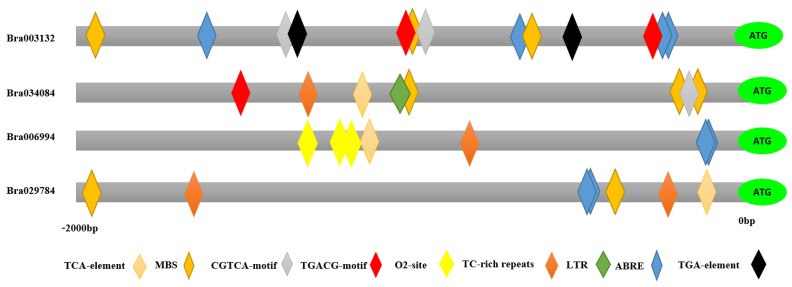
Cis-element analysis of the S1fa genes in Chinese cabbage. Different colors represent different cis-elements in the promoter region of the S1fa family genes in Chinese cabbage.

Cis-elements are the regions of non-coding DNA that regulate the transcription of the neighboring genes. The cis-elements of Chinese cabbage S1fa genes were identified in the promoter region as presented in Figure 3. The results show that the *Bra034084* gene is located on chromosome A07, which has 1 GATA-motif, 1 LTR, 1 TC-rich repeat, 1 TCA-element, 1 CGTCA-motif, 1 GT1-motif, 1 TGACG-motif, 2 AE-box, 3 AREs and 3 TCT-motifs. *Bra003132* has 1 LTR, 1 MBS, 1 AE-box, 1 CAT-box, 1 TCCC-motif, 1 TCT-motif, 1 LAMP-element, 2 CGTCA-motifs, 2 TGACG-motifs, 2 TGA-elements, 3 AREs, 3 MBSs, 4 ABREs and 6 G-boxes (Supplementary Table S1). *Bra006994* has 1 LTR, 1 TCA-element, 1 ARE, 1 Box II, 1 CAT-box, 1 TC-rich repeats, 2 TCT-motifs, 2 ABREs, 2 TCT-motifs and 3 O_2_-sites. *Bra029784* has 1 TCA-element, 1 ARE, 1 AE-box, 1 G-box, 1 Box-II, 1 *chs*-Unit 1 m1, 2 TC-rich repeats, 2 MBSs, 2 ABREs and 2 TCT-motifs, which are involved in facilitating a plant’s physiological and biochemical mechanisms under abiotic stresses.

### 2.4. Structure and Motif Analysis of the S1fa Genes

**Figure 4:**
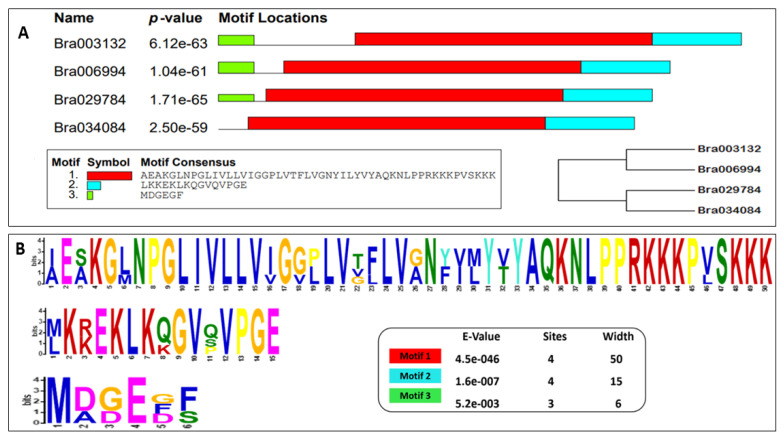

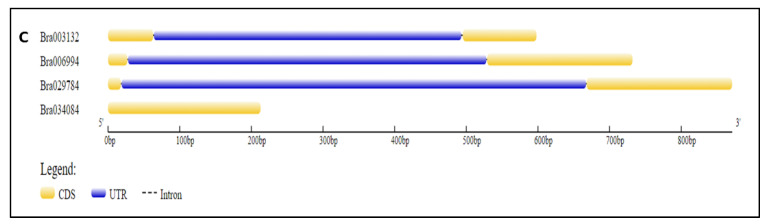
Analysis of motifs and gene structures of S1fa proteins in Chinese cabbage. (**A**) Protein motifs, location and phylogenetic trees in the S1fa family members. (**B**) The sequence of three identified motifs in Chinese cabbage. (**C**) Gene structure of the S1fa family member in Chinese cabbage.

To explore the features of the S1fa genes, the conserved motifs of the genes in Chinese cabbage were analyzed. The results show that S1fa consists of three common motifs, namely, motif 1, 2 and 3, as presented in Figure 4. Motif 1 is the largest motif with a length of 55 aa, which is localized in the middle of the S1fa gene, followed by motif 2 and motif 3, respectively. Similarly, the exon–intron structures of the S1fa genes were analyzed (Figure 4). *Bra006994*, *Bra003132* and *Bra029784* have the same structure, while *Bra034084* has a different structure. The coding sequence of *Bra006994*, *Bra003132* and *Bra029784* is localized on the left and right borders of the UTR, and shares a similar gene structure, but *Bra034084* does not have a UTR.

### 2.5. Expression Profiles of the S1fa Genes in Different Tissues

**Figure 5:**
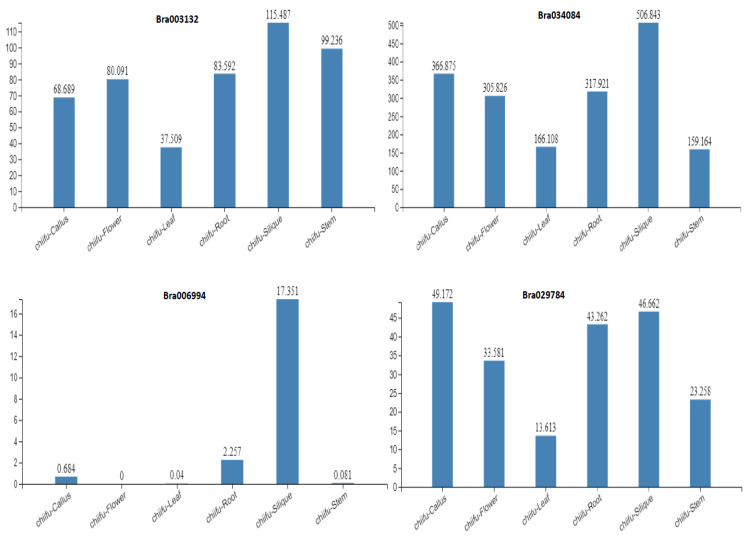
The transcription levels of the S1fa genes in different tissues, including root, leaves, flower, stem, callus and silique of Chinese cabbage. Data were obtained from Chinese cabbage database.

To explore the potential functions of the S1fa genes in growth and development, the tissue-specific characteristics were obtained from the Chinese cabbage database (http://brassicadb.cn/#/ (accessed on 22 March 2022)). The results show that the TPM (Transcript per million) values of the S1fa genes varied in different plant tissues. The S1fa genes were highly expressed in the silique of Chinese cabbage (Figure 5), while the expression level was the lowest in the leaf tissue. Comparative analyses of the S1fa genes show that *Bra034084* had the highest expression, followed by *Bra003132* and *Bra006994*, respectively. *Bra029784* had the least expression in all tissues except the silique tissues compared with other members of the S1fa genes. Moreover, the expression of the S1fa genes was downregulated in the leaf and flower tissues, while *Bra006994* showed no expression in the leaf and flower tissues. Taken together, these findings indicate that the S1fa genes are actively expressed in Chinese cabbage, which could play vital roles in Chinese cabbage growth and developmental process. Thus, it is necessary to investigate the functions of the S1fa genes in abiotic stress tolerance.

### 2.6. Expression Patterns of the S1fa Genes under Abiotic Stress

**Figure 6:**
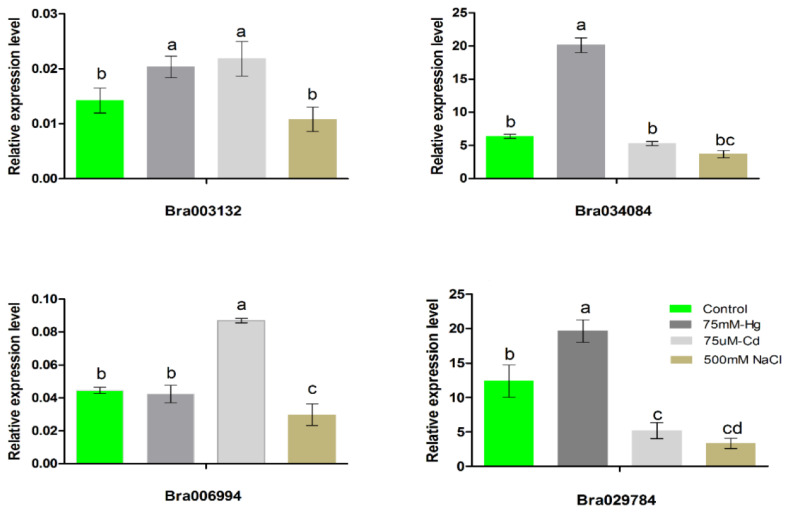
The expression levels of the S1fa genes under NaCl, Cd and Hg stresses. Different colors indicate different stresses and the letters above error bars represent significant differences at *p* > 0.05.

The S1fa transcription factor plays an important role in regulating plant growth and development, and abiotic stress tolerance. However, the involvement of the S1fa genes in response to abiotic stresses is not clear. To confirm the molecular mechanism of the S1fa genes in response to abiotic stresses, their transcript abundance was investigated under Hg, Cd and NaCl stresses (Figure 6). The expression levels of all four S1fa genes were investigated 24 h after the stress treatments. The S1fa genes were significantly expressed under abiotic stresses. Under Hg stress, the expressions of the *Bra034084* and *Bra029784* genes were significantly elevated compared with the control (CK) treatment, followed by *Bra003132*. Likewise, under Cd stress, the *Bra003132* and *Bra006994* genes showed high expression levels compared with CK, while NaCl stress significantly reduced the expression of the S1fa genes (Figure 6). These findings suggest that the S1fa genes are involved in and positively induced by various abiotic stresses in Chinese cabbage.

### 2.7. Prediction of miRNAs Targeting the S1fa Genes in Chinese Cabbage

miRNAs are a class of non-coding single strand RNA molecules of approximately 22 nucleotides, which are encoded by endogenous genes and may be involved in the activation of genes in response to abiotic stresses. A total of 39 miRNAs targeting the S1fa genes in Chinese cabbage are presented in Table 2, including 5 miRNAs targeting *Bra003132* (*ath-miR5661*, *ptc-miR397c*, *mtr-miR2641*, *hvu-miR6214* and *hme-miR-278*), 11 miRNAs targeting *Bra006994* (ptc-*miR397c*, *zma*-*miR399e-5p*, *aly-miR160c*-*3p*, *ath-miR5661*, *osa-miR160a-3p*, *osa-miR160b-3p*, *zma-miR160b-3p*, *zma-miR160g-3p*, *bdi-miR160b-3p, bdi-miR160c-3p* and *ata-miR160c-3p*), 11 miRNAs targeting *Bra029784* (*aly-miR838-3p*, *zma-miR399e-5p*, *ath-miR838*, *osa-miR3982-3p*, *bdi-miR398b, osa-miR2095-3p*, *aly-miR4248a*, *aly-miR4248b*, *aly-miR4248c*, *gma-miR4363* and *bdi-miR7757-3p.1*) and 12 miRNAs targeting *Bra034084* (*aly-miR838-3p*, *zma-miR399e-5p*, *ath-miR838*, *mtr-miR2673a*, *mtr-miR2673b*, *gma-miR4363*, *bdi-miR398b*, *osa-miR2055*, *osa-miR3982-3p*, *cca-miR6116-3p*, *stu-miR8050-3p* and *gma-miR9752*).

### 2.8. S1fa Overexpression in Response to Abiotic Stresses in Yeast

**Figure 7:**
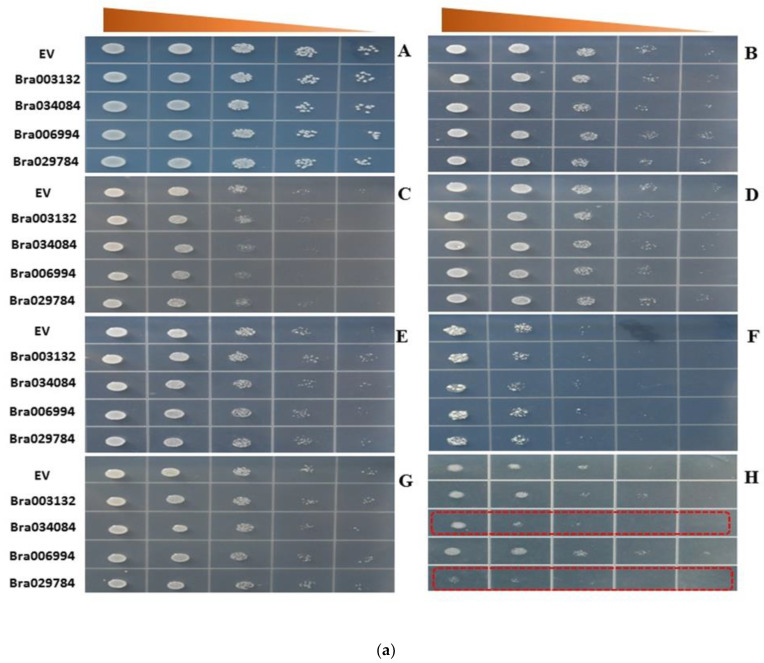

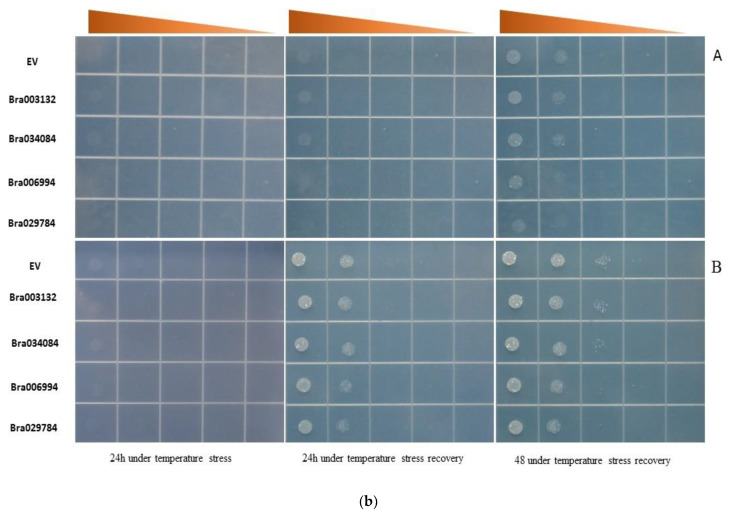
(**a**) Responses to abiotic stresses through yeast dilution bioassay with the wild type strain, S1fa transformed with pRS-416-GFP. Yeast wild type (EV) strain and S1fa overexpressing cells were grown in the URA liquid medium for 24 h at 28 °C. The cell solutions were diluted to an OD_600_ value of 0.3 and exposed to different types of abiotic stresses. (**A**) URA (control), (**B**) 50 mM Cu, (**C**) 75 mM Hg, (**E**) 100 mM Al, (**D**) 100 mM Co, (**F**) 75 μM Cd, (**G**) 2 M mannitol, (**H**) 1 M NaCl. Triangles represent the 10-fold serial dilutions (the starting OD_600_ is 0.3). (**b**) Responses to temperature stress tolerance through yeast dilution bioassay with the wild type strain, S1fa transformed with pRS-416-GFP. The yeast cells were exposed to (**A**) low temperature (4 °C) and (**B**) high temperature (38 °C) for 24 h, and then transferred to a place with the normal temperature (28 °C). Triangles represent the 10-fold serial dilutions (the starting OD_600_ is 0.3).

To elucidate the function of the S1fa genes in abiotic stress tolerance, we generated an overexpression model of yeast using the pRS416 vector. The yeast cells with S1fa overexpression were exposed to abiotic stresses (75 μM-Cd, 75 mM-Hg, 100 mM-Al, 50 mM-Cu, 100 mM-Co, 1M-NaCl, 2M-Mannitol, and cold and heat stresses (24 h stress followed by two days with the normal temperature)) (Figure 7a(A,B)). The overexpression of *Bra034084*, *Bra003132*, *Bra029784* and *Bra006994* and EV (empty vector) showed no effects on Al, Mannitol, Co and cold stresses, However, the cells were sensitive to NaCl, Hg and Cd stresses, compared with EV (Figure 7a(C,F,H)). Under NaCl stress, *Bra006994* and *Bra029784* were highly sensitive, when compared to EV (Figure 7a(H)). Likewise, under heat stress (38 °C) and cold stress (4 °C), all cells with the overexpression of Chinese cabbage S1fa genes grew slightly slower than EV (Figure 7b(A,B)). Cells overexpressed with *Bra006994* and *Bra029784* were more sensitive to these stresses (Figure 7b(A,B)). These results indicate that the *Bra006994* and *Bra029784* genes play an important role in response to salt stresses.

### 2.9. Growth Curve

**Figure 8:**
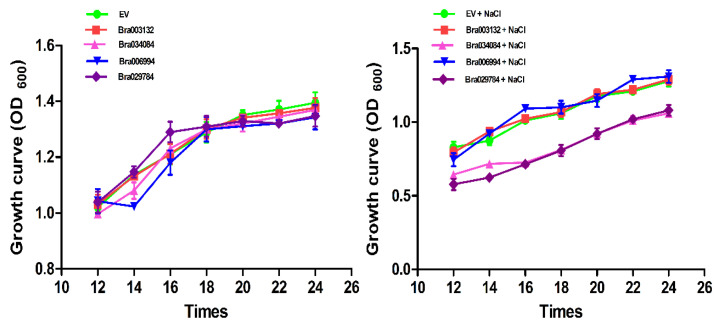
Growth curves of the S1fa gene overexpressing yeast cells under 1 M NaCl stress. EV (empty vector (yeast WT)) and cells with the overexpression *of Bra003132, Bra006994, Bra034084* and *Bra029784* were grown at 28 °C. Cell density was monitored after 12, 14, 16, 18, 20, 22 and 24 h after the treatment. The error bar represents the deviation of three independent replications.

To confirm the responses of the S1fa-overexpressing yeast cells to salinity, we conducted the growth curve of the yeast cells under salt stresses. The yeast cells were incubated under 28 °C until OD_600_ reached 0.3, and then were treated with 1 M NaCl in liquid URA medium. The OD_600_ values were observed after 12, 14, 16, 18, 20, 22 and 24 h, respectively. The results indicate that *Bra034084* and *Bra029784* overexpressing yeast cells were highly sensitive to NaCl stresses (Figure 8). Under normal conditions, the growth rate of the S1fa expressing cells was the same as that of EV, whereas under NaCl stresses, the optical density was significantly decreased compared with that of EV. The growth rate of the *Bra003132* and *Bra006994* overexpressing yeast cells showed no difference compared with that of EV, but was significantly higher than those of the *Bra034084* and *Bra029784* overexpressing yeast cells (Figure 8). These findings suggest that salinity stresses had detrimental effects on the growth of the *Bra034084* and *Bra029784* overexpressing yeast cells.

### 2.10. Subcellular Localization of the S1Fa Genes

Subcellular localization is considered a key parameter for transcription factor responses to abiotic stresses. However, no evidence was found to support the translocation of the S1fa genes into the nucleus under salt stresses.

**Figure 9:**
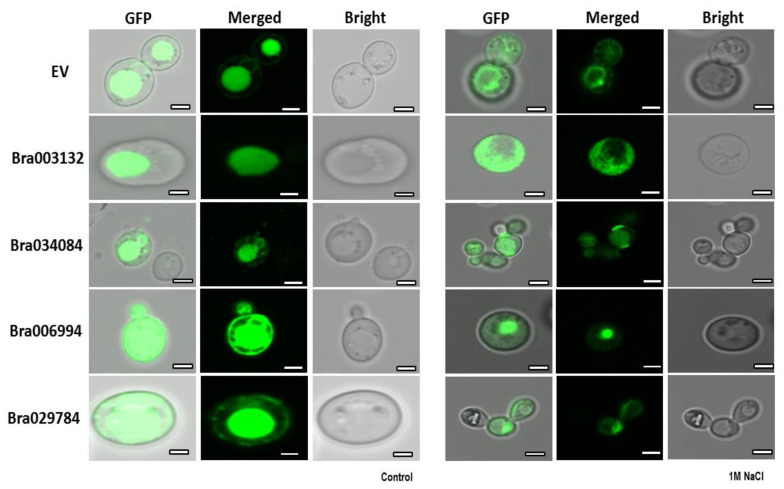
The subcellular localization of the S1fa genes and the empty vector (EV) tagged with GFP and transiently expressed in yeast cells treated with 1 M NaCl. The images were obtained from GFP, merged and bright channels. Scale bar: 10 μm.

To confirm the subcellular localization, the S1fa genes were transiently expressed in the fusion of GFP in yeast, and the fluorescence was observed using a confocal microscopy. Without salt stress, the S1fa genes were observed in the nucleus as dot-like structures as presented in Figure 9. When treated with salt, *Bra034084* and *Bra029784* were translocated into the cell wall.

### 2.11. Responses of Cell Wall Biosynthesis Genes to NaCl Stresses

**Figure 10:**
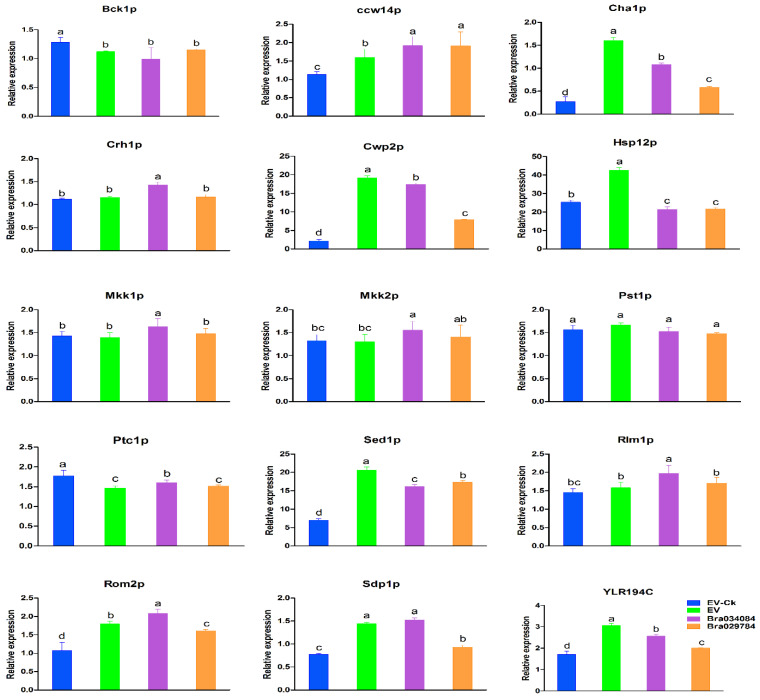
Quantitative reverse transcription polymerase chain reaction (qRT-PCR) analysis was used to assess the expression levels of cell wall biosynthesis genes under NaCl stresses. The error bar represents the deviation of three independent replications. The letters above the error bar represent significant differences at *p* > 0.05.

In eukaryotic organisms, the biological integrity depends on the cell wall, which is considered an essential structure that not only maintains the morphology, but also participates in protecting the cell from environmental influences. Here, in this study, we investigated the expression levels of cell wall biosynthesis genes as presented in Figure 10. The results suggest that the S1fa genes *Bra034084* and *Bra029784* significantly enhanced the expression levels of cell wall biosynthesis genes compared with EV-Ck (no stress) and EV (with stress). The expression levels of cell wall biosynthesis genes, including *Ccw14p*, *Cha1p*, *Cwp2p*, *Sed1p*, *Rlm1p*, *Rom2p*, *Mkk1p*, *Hsp12p*, *Mkk2p*, *Sdp1p* and *YLR194c*, were significantly enhanced, while the expression levels of *Bck1p* and *Ptc1p* were downregulated under salt stresses in *Bra034084* and *Bra029784* overexpressing yeast cells. The expression of the *Pst1p* gene showed no significant difference. These findings suggest that *Bra034084* and *Bra029784* activate the signaling pathways regulating cell wall integrity by stimulating transcriptional and post-transcriptional genes in response to salt stresses.

### 2.12. Antioxidant Enzyme Activities and ROS Accumulation under NaCl Stresses

**Figure 11:**
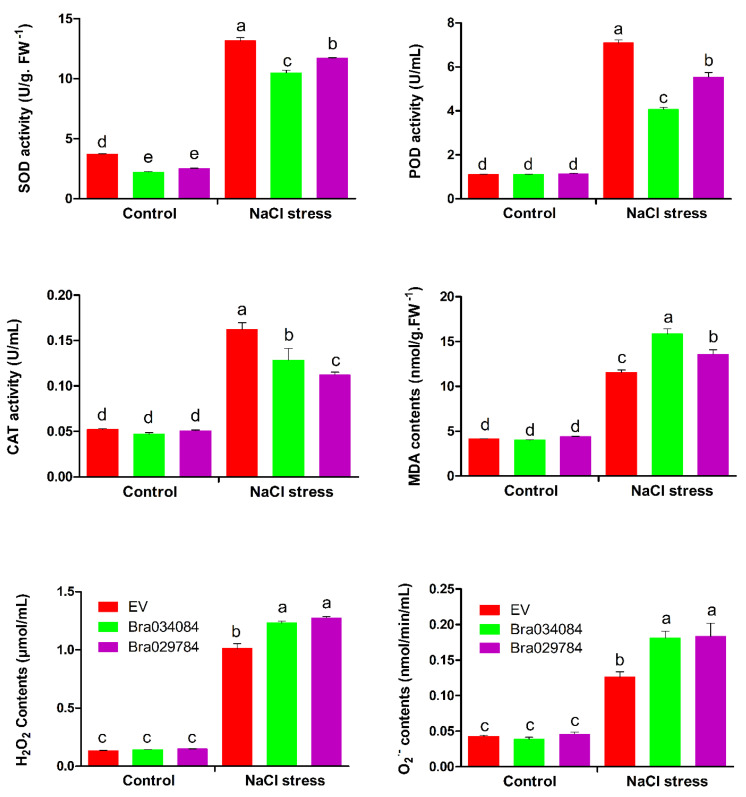
The effect of salt stress on antioxidant enzyme activities, MDA and ROS accumulations of Bra034084 and Bra029784 overexpressing yeast cells. *Bra034084* and *Bra029784* overexpressing cells were exposed to 1 M salt stress and control (without stress) for 14 h at 28 °C. Superoxide dismutase (SOD), peroxidase (POD), catalase (CAT), malondialdehyde (MDA), hydrogen peroxide (H_2_O_2_), and superoxide radicals (O_2_^−^). Different letters indicate significant differences at *p* < 0.05.

The antioxidant enzyme activities, the MDA content and the ROS accumulations in the *Bra034084* and *Bra029784* overexpressing cells were analyzed after 14 h of exposure to NaCl stresses. The results suggest that the antioxidant enzyme activities were greatly reduced by NaCl stresses compared with EV, as presented in Figure 11. The activities of SOD and POD were reported to be significantly higher in the *Bra029784* overexpressing cells than those in the *Bra034084* overexpressing cells, whereas the activity of CAT was enhanced in the *Bra034084* overexpressing cells. Moreover, the activities of CAT, SOD and POD were significantly higher under NaCl stress than those in the control. The MDA content was higher in the *Bra034084* overexpressing cells, while the contents of H_2_O_2_ and O_2_^−^ were not affected but were significantly higher than those of EV. The contents of ROS and MDA of NaCl stress were significantly higher than the control, as presented in Figure 11. These findings suggest that salinity stress negatively affected the activities of antioxidant enzymes but promoted the accumulation of ROS and MDA, which may activate the biosynthesis of the cell wall, and hence increase salt stress sensitivity.

## 3. Discussion

Environmental influence impairs plant growth and development through oxidative stresses, which may change genome stability [10,20]. Plants have evolved a number of pathways to cope with ROS production and reduce the detrimental effects of abiotic stresses, including antioxidant enzymes, hormonal responses, the activation of transcription factors [8] and regulation of downstream genes [9,21].

Transcription factors (such as MYB, WRKY, ERF and NAC) play a crucial role in abiotic stress tolerance, and are involved in the transcriptional regulation of plant genes [22,23,24,25]. The S1fa transcription factor belongs to the smallest family of plants that are involved in plant growth and development [18]. However, no study has reported on their regulatory effects on abiotic stresses in Chinese cabbage. The present study was designed to investigate the physiological and molecular mechanism of the S1fa genes in abiotic stress tolerance. We identified and characterized four S1fa proteins in Chinese cabbage at the whole genome level, which were also compared with three *Arabidopsis* S1fa proteins. Systematic analyses including phylogenetic trees, gene and protein structures, motifs, physiochemical properties, miRNAs and cis-elements were conducted in the promoter region of the S1fa genes, and the effects of the S1fa genes on abiotic stress tolerance were investigated in yeast models. These findings provide novel insights into the functional characterization of the S1fa genes, which can be used in molecular breeding to enhance crop production and abiotic stress tolerance.

Based on phylogenetic analyses, the S1fa proteins were divided into three groups. Chinese cabbage S1fa proteins were classified in groups I and II, which share a high similarity with those in rice, pepper, cucumber and watermelon (Figure 2). The difference between these groups demonstrates that these proteins underwent great genetic variation after divergence, probably due to environmental influences, and that some gene fragments might have been lost during the evolutionary process [26]. The analysis of gene structure and motif suggested that the S1fa genes shared a similar exon–intron and motif structure, indicating there is a closer evolutionary relationship among the members in the same group but a functionally diversified relationship among the other group members [27,28,29,30]. The structure and motif analysis of Chinese cabbage S1fa genes showed a similar structure, which has three common motifs (Figure 4). Interestingly, based on motif analysis, the S1fa genes in Chinese cabbage can be divided into two subgroups, with *Bra003132* and *Bra006994* in one group, and *Bra029784* and *Bra034084* in the other (Figure 4). Protein analysis shows that motif 1 is highly conserved, which may be involved in or required for recognizing abiotic stress responsive cis-elements in response to stresses [31,32,33].

A gene’s expression pattern can provide important indications for its biological functions. In the current study, the expression patterns of the S1fa genes were analyzed under abiotic stresses in Chinese cabbage (Figure 6). The S1fa genes showed different expression patterns in different tissues. The S1fa genes had the lowest expression in the leaves and the highest expression in the silique (Figure 5). *Bra006994* showed a minimum expression level in the leaf, flower and callus. The expression level of the S1fa genes was significantly different under different abiotic stresses (Figure 6). Under NaCl stress, *Bra034084* and *Bra029784* were significantly expressed compared with the other two S1fa genes. However, their expressions were significantly downregulated under Cd and Hg stresses (Figure 6). Taken together, these results suggest that the S1fa transcription factors are potentially involved in salinity stress tolerance, as well as plant growth and development [17,18].

The cis-element is a specific sequence on the promoter region of a given gene, which influences the expression of protein-coding and long non-coding RNA genes. Activation of the expression of the gene by binding with the cis-element is a common way of regulating developmental and physiological processes [33]. In plants, miRNAs act as a positive regulator in regulating related genes. Many studies have shown that miRNAs are involved in response to abiotic stresses [34]. *miR398b* negatively regulates the defense system in cotton and causes an adverse effect on plant growth. *miR1885* regulates plant growth and tolerance to viral infection through targeting *BraTNL1* and *BraCP24* genes in *Brassica* [35]. *Bra034084* and *Bra029784* were targeted by miRNAs including miR398b and miR1885, which might be involved in salt stress responses (Figure 7a and Figure 7b). Under abiotic stresses, the cis-elements are involved in controlling the transcriptional regulation of the core gene network [31]. The S1fa genes of Chinese cabbage contain a number of cis-elements including light and abiotic stress responsive elements, ABA, GA, methyl jasmonate (MeJA), and low and high temperature responsive elements (Figure 3). Previous studies have reported that hormonal cis-elements, such as O_2_-site, TGA-element, TGACG-motif, CGTCA-motif, TCA-element and ABRE motifs, are the key cis-regularity modules that stimulate the hormone signaling pathways under abiotic stresses [31,33]. The cis-elements activate specific transcription factors and their downstream genes, acting as a key cellular regulator in response to abiotic stresses [33]. These cis-elements may also be involved in salinity stress tolerance by regulating the specific hormonal signal transduction pathways [33].

The S1fa transcription factors have been reported to be involved in photomorphogenesis and abiotic stress tolerance [17]. However, their function has yet to be fully understood. Here, four Chinese cabbage S1fa genes were identified and cloned to investigate their function in abiotic stress tolerance using a yeast model (Figure 7a and Figure 7b). The results suggest that the *S1fa* genes did not respond to any abiotic stress. However, *Bra034084* and *Bra029784* were highly sensitive to NaCl stress (Figure 7a(H)), suggesting that the S1fa transcription factors are involved in salinity stress tolerance. These findings are in line with a previous study, which shows that *OsS1fa* improves drought stress tolerance in *Arabidopsis* and increases the expression of the drought stress-related genes [18].

Plants exposed to abiotic stresses generate an excessive amount of ROS (H_2_O_2_ and O_2_^−^), which is highly toxic and detrimental to proteins, lipids, DNA and carbohydrates, eventually leading to cell death [6,36,37]. The plant possesses an antioxidant enzyme defense system to normalize the overproduction of ROS. In this study, the overexpression of *Bra034084* and *Bra029784* significantly decreased the activities of antioxidant enzymes including SOD, POD and CAT under salinity stresses in yeast (Figure 11). On the other hand, the overexpression of *Bra034084* and *Bra029784* enhanced the accumulation of H_2_O_2_, O_2_^−^ and MDA in the *S1fa* overexpressing yeast cells compared with the wild type (EV) (Figure 11). These findings suggest that *Bra034084* and *Bra029784* inhibit the antioxidant enzyme activities, thereby leading to a higher hypersensitivity to salt stresses. *PsS1Fa2* overexpression in *Populus trichocarp* enhanced drought stress tolerance by increasing the activities of antioxidant enzymes (SOD and POD) and reducing the accumulation of MDA, H_2_O_2_ and O_2_^−^ [17]. A similar report has been conducted, which shows *CaDHN4* can protect against cold and salt stresses by activating the antioxidant enzyme defense system [38].

In eukaryotic organisms, the cell wall plays a dominant role in the protection of the cell from environmental influences [11]. In yeast, transcriptional re-programing can alter the expression of key genes for cell wall biosynthesis, energy generation, signal transduction and stress [11,39]. Several signaling pathway cascades such as *MAPK*, *MAPKK1*, *MAPKK2* and *Slt2* have been reported to be involved in cell wall biosynthesis [40,41]. In this study, the overexpressed S1fa genes, *Bra034084* and *Bra029784*, were located in the cytoplasm, and were translocated under NaCl stresses (Figure 7a and Figure 7b), and they significantly enhanced the expression level of cell wall biosynthesis genes (Figure 11). The *PKC1* (protein kinase C) pathway plays an important role in cell wall biogenesis, maintenance and cell integrity [41], and is regulated by *Bck1p*, *Mkk1p*, *Slt2p* and *Rom2p* [11,41]. Our study showed that the expression of these factors was significantly increased in the S1fa overexpressing yeast cells under salinity stresses, as presented in Figure 10. Additionally, the overexpression of *Bra034084* and *Bra029784* enhanced the transcript level of *Bck1p*, *Ptc1p Ccw14p*, *Crh1p*, *Mkk1p*, *Mkk2p*, *Rlm2* and *Rom2*, which are involved in the signaling pathway regulating cell wall integrity [11,39,40]. Subcellular localization analysis suggests that the S1fa gene was translocated from the cytoplasm to the cell wall under salt stresses, as presented in Figure 9, which in turn might promote the expression of many cell wall biosynthesis genes. These findings are consistent with previous studies, which show that abiotic stress induces a significant reduction in the activities of antioxidant enzymes and increases the contents of ROS and MDA [11,14], thereby regulating the expression of the genes controlling cell wall integrity [11]. In summary, it can be concluded that the S1fa transcription factors activate genes involved in cell wall integrity under salinity stresses. Future studies are required to investigate the linkage between cell wall integrity and S1fa genes under abiotic stresses, especially salinity stresses.

## 4. Materials and Method

Chinese cabbage (Cv. Guangdongzao) seeds were soaked with 1% sodium hypochlorite for 3 min and then washed at least five times with ddH_2_O to remove the excessive sodium hypochlorite. Then, the seeds were germinated in ½MS media in a controlled growth chamber as described previously [42]. The uniform seedlings were transferred to a hydroponic culture and incubated for five more days before treated with 75 μM Cd, 75 mM Hg and 1 M NaCl, respectively. The samples were collected and ground in liquid nitrogen to extract the total RNA [43].

### 4.1. Identification of the S1fa Genes in Chinese Cabbage

In order to identify the S1fa genes in Chinese cabbage, the protein sequences of three S1fa genes from TAIR (www.Arabidopsis.org (accessed on 22 March 2022)) were downloaded and used as queries in Chinese cabbage genome database (http://brassicadb.cn (accessed on 22 March 2022)) with the BLASTP program [42]. All predicted Chinese cabbage S1fa proteins were confirmed through Pfam (http://pfam.xfam.org/ (accessed on 22 March 2022)) and SMART database. The physicochemical parameters including protein isoelectric point (pl), molecular weight (kDa) and length were calculated using the tools on the ExPASy server (http://web.expasy.org/compute_pi/ (accessed on 22 March 2022)). The chromosomal locations and strand directions were obtained from the BRAD database (http://brassicadb.cn (accessed on 22 March 2022)), and the subcellular location of each protein was investigated using CELLO 2.5 (http://cello.life.nctu.edu.tw/ (accessed on 22 March 2022)) [29].

### 4.2. Phylogenetic Trees and Sequence Alignment

The full length protein sequences from Chinese cabbage, tomato, pepper, cotton, *Arabidopsis*, cucumber, watermelon and rice were obtained from the genome database and aligned using MUSCLE (https://www.ebi.ac.uk/Tools/msa/muscle (accessed on 22 March 2022)), which were used to construct the evolutionary tree using the MAGA7 software by the neighbor-joining method, and protein sequence alignment was performed as described previously [29].

### 4.3. S1fa Structure and Conserved Motif

The S1fa gene structure was analyzed using the online MEME program (http://meme-suite.org/tools/meme(accessed on 22 March 2022)), and the maximum number of motifs was set at 10. The structures of the S1fa genes were designed by using the online program GSDS 2.0 (http://gsds.cbi.pku.edu.cn/ (accessed on 22 March 2022)) [29]. The online tool PlantCARE (http://bioinformatics.psb.ugent.be/webtools/plantcare/html/ (accessed on 22 March 2022)) was used to analyze the cis-elements as described previously [28,29,44].

### 4.4. S1fa Gene Promoter Analysis and miRNA Prediction

The promoter sequences of Chinese cabbage S1fa genes (2000 bp upstream) were obtained from Chinese cabbage genome database (http://brassicadb.cn (accessed on 22 March 2022)). The online tool PlantCARE (https://bioinformatics.psb.ugent.be/webtools/plantcare/html/ (accessed on 22 March 2022)) was used to analyze the cis-elements in the promoter sequence [17]. To predict the miRNAs, the coding sequence of the S1fa genes was submitted to the psRNATarget server (https://www.zhaolab.org/psRNATarget/ (accessed on 22 March 2022)) as the targeted candidate as described previously [32].

### 4.5. Total RNA Extraction and qRT-PCR Analysis

The total RNA was extracted for Chinese cabbage tissues using TRIzol, while yeast RNA was extracted using the M5 EASYspin yeast RNA rapid extraction kit, MF158-01 (Mei5 Biotechnology, Co., Ltd. Beijing China). For yeast RNA extraction, the cells were grown until the OD_600_ value reached 0.3 at 28 °C, and then treated with 1 M NaCl for 12 h before the total RNA was harvested [45]. The first-stand cDNA was synthesized using a PrimeScript and RT reagent kit with gDNA Eraser (TAKARA). The SYBR Premix Ex-Taq Kit (TAKARA) was used for quantitative real-time PCR. All experiments were performed with three independent biological replications. The transcript levels were calculated using the 2^∆∆^-CT method. The TMP values of Chinese cabbage tissues were obtained from the Chinese cabbage database (http://brassicadb.cn (accessed on 22 March 2022)) for each S1fa gene. The primers used for qRT-PCR are presented in Supplementary Table S1.

### 4.6. Yeast Constructs

To construct the yeast (*Saccharomyces cerevisiae*) overexpression vectors, the coding sequences of Chinese cabbage genes, *Bra034084*, *Bra003132*, *Bra029784* and *Bra006994*, were cloned separately into the pRS-416-GFP vector. The coding sequences of the S1fa genes were amplified from Chinese cabbage cDNA with specific primers (Supplementary Table S1) and then inserted into the SPE1 site on pRS-416-GFP using the infusion cloning kit (Catalog no. 011614; Clontech) [46]. The sequence insertions were confirmed through SANGER sequencing and then used for the investigation of abiotic stress tolerance in yeast. To determine the subcellular localization of the S1fa proteins, the S1fa genes were inserted into pRS-416-GFP. The subcellular localization of the fusion proteins was observed under a Zeiss Axiophot fluorescence microscope as described previously [45].

### 4.7. Tolerance Assay and Growth Curve

The final pRS-416-GFP vectors cultured in URA medium were diluted until the OD_600_ value was 0.1, and were incubated again until the OD_600_ reached 0.3. The cell culture was then five-fold diluted and treated with 75 μM Cd, 75 mM Hg, 100 mM Al, 50 mM Cu, 100 mM Co, 1 M NaCl and 2 M mannitol, respectively, and was incubated at 28 °C for five days [45]. No treatment was added for the control. The cold and heat stresses were applied at 4 °C and 38 °C, respectively, for 2 days before being transferred to a place at 28 °C to for 3 more days. The photos were taken after five days of incubation, and the experiment was repeated three times. The S1fa overexpressing yeast cells without and with the 1 M NaCl treatment were grown at 28 °C in liquid URA culture medium, and diluted until the OD_600_ value was about 0.1. The cells were incubated again, and when OD_600_ value reached 0.3, the OD_600_ was recorded every 2 h to prepare the growth curve of the cells [45].

### 4.8. Determination of Antioxidant Enzyme Activities and ROS Contents

To determine the antioxidant enzyme activities and ROS contents, the yeast cells were harvested 14 h after the NaCl treatment at 28 °C and stored at −80 °C. The antioxidant enzyme activities and ROS and MDA contents were analyzed using the service provided by Nanjing Ruiyuan Biotechnology company (https://bestofbest.top/).

## 5. Statistical Analysis

Three independent biological replications were used for each treatment, and the whole experiment was repeated three times. The data were statistically analyzed using an analysis of variance, and compared with the control using the LSD test (*p* > 0.05) by using the Statistix 8.1 software (https://www.statistix.com/). The Graphpad Prism 5 software was used for graphical presentation.

## 6. Conclusions

In this study, four S1fa family proteins were identified in Chinese cabbage, and the chromosomal location, structure, phylogenetic tree and physiochemical properties were analyzed, and molecular characterization performed in yeast, to understand their involvement in abiotic stress tolerance. The S1fa proteins have three highly conserved motifs. Moreover, the cis-elements and miRNAs targeting S1fa genes were predicted to understand the regulatory mechanism. The expression patterns of the S1fa gene in different tissues and their responses to abiotic stresses show that these genes may play a significant role in the growth and development of Chinese cabbage. In yeast, *Bra034084* and *Bra029784* was highly sensitive to salt stresses and might activate the expression of cell wall biosynthesis genes. For the first time, we elucidate the functions of the S1fa genes in Chinese cabbage and confirm their responses to salt stresses. The comprehensive understanding of the physiological and molecular mechanisms of *Bra034084* and *Bra029784* can serve as an important genetic resource for the improvement in salinity stress tolerance and the yield of Chinese cabbage.

## Figures and Tables

**Table 1:** The characteristics of the S1fa genes in Chinese cabbage.

Gene ID	Protein Length (aa)	Molecular Weight (KD)	Chromosome Location	pl	Strand Direction	Subcellular Location
***Bra003132***	88	9.3	A07: 14791441..14792038	10.06	-	Nuclear
***Bra034084***	70	7.8	A01: 25080494..25080706	10.38	+	Nuclear
***Bra006994***	76	8.3	A09: 28232711..28233442	10.05	-	Nuclear
***Bra029784***	73	8.1	A05: 23013673..23014543	10.06	+	Nuclear

**Table 2:** Prediction of miRNAs targeting the S1fa genes of Chinese cabbage.

miRNA	Target S1fa	Target Site	miRNA Fragment	Alignment	Target Fragment	Inhibition
**ath-miR5661**	***Bra003132***	146–166	AGAGGUACAUCAUGUAGUCUG	**:::::.:::::::: : :**	CCAACUACGUGAUGUACGUGU	Cleavage
**ptc-miR397c**	***Bra003132***	114–134	UCAAUGAGUGGAGCUUUGAUG	**..::..:: :::.::..:::**	UGUCGGAGGUCCGCUUGUUGU	Cleavage
**mtr-miR2641**	***Bra003132***	71–90	GUUUGAUCCUUUACGUUUAU	**:.:: :::::::..:::**	CUGAAGCCAAAGGAUUGAAC	Cleavage
**hvu-miR6214**	***Bra003132***	99–118	CGACGACGACGAGCACGACA	**::::::: :.::.::::**	AAUCGUGCUGCUUGUUGUCG	Translation
**hme-miR-278**	***Bra003132***	3–23	UCGGUGGGAUCUUCGUCCGUUU	**..:.::: ::::.:..:.:::.**	GGAUGGA-GAAGGUUUCGCCGG	Cleavage
**ptc-miR397c**	***Bra006994***	78–98	UCAAUGAGUGGAGCUUUGAUG	**.:::..::.::::::..:::.**	UAUCGGAGUUCCACUUGUUGG	Cleavage
**zma-miR399e-5p**	***Bra006994***	148–168	GGGCUUCUCUUUCUUGGCAGG	**::: : :.:::. ::::::::**	CCUCCAAGGAAGAAGAAGCCC	Cleavage
**aly-miR160c-3p**	***Bra006994***	91–110	GCGUACAAGGAGCCAAGCAUG	**: ::.: :::::::::: :::**	CUUGUUGGGCUCCUUGU-CGC	Cleavage
**ath-miR5661**	***Bra006994***	110–130	AGAGGUACAUCAUGUAGUCUG	**:::::.:::::::: : :**	CCAACUACGUGAUGUACGUGU	Cleavage
**osa-miR160a-3p**	***Bra006994***	91–110	GCGUGCAAGGAGCCAAGCAUG	**: ::.: :::::::::. :::**	CUUGUUGGGCUCCUUGU-CGC	Cleavage
**osa-miR160b-3p**	***Bra006994***	91–110	GCGUGCAAGGAGCCAAGCAUG	**: ::.: :::::::::. :::**	CUUGUUGGGCUCCUUGU-CGC	Cleavage
**zma-miR160b-3p**	***Bra006994***	91–110	GCGUGCAAGGAGCCAAGCAUG	**: ::.: :::::::::. :::**	CUUGUUGGGCUCCUUGU-CGC	Cleavage
**zma-miR160g-3p**	***Bra006994***	91–110	GCGUGCAAGGAGCCAAGCAUG	**: ::.: :::::::::. :::**	CUUGUUGGGCUCCUUGU-CGC	Cleavage
**bdi-miR160b-3p**	***Bra006994***	91–110	GCGUGCAAGGAGCCAAGCAUG	**: ::.: :::::::::. :::**	CUUGUUGGGCUCCUUGU-CGC	Cleavage
**bdi-miR160c-3p**	***Bra006994***	91–110	GCGUGCAAGGAGCCAAGCAUG	**: ::.: :::::::::. :::**	CUUGUUGGGCUCCUUGU-CGC	Cleavage
**ata-miR160c-3p**	***Bra006994***	91–110	GCGUGCAAGGAGCCAAGCAUG	**: ::.: :::::::::. :::**	CUUGUUGGGCUCCUUGU-CGC	Cleavage
**aly-miR838-3p**	***Bra029784***	161–181	UUUUCUUCUUCUUCUUGCACA	**: : ::::::::::: :::.:**	UUUCCAAGAAGAAGAUGAAGA	Cleavage
**zma-miR399e-5p**	***Bra029784***	139–159	GGGCUUCUCUUUCUUGGCAGG	**::: : :.:::. ::::::::**	CCUCCGAGGAAGAAGAAGCCC	Cleavage
**ath-miR838**	***Bra029784***	161–181	UUUUCUUCUACUUCUUGCACA	**: : ::::::: ::: :::.:**	UUUCCAAGAAGAAGAUGAAGA	Translation
**osa-miR3982-3p**	***Bra029784***	86–106	AGUUGCCUACAUGGAGCGCCA	**:: ::.::: :::::: ::::**	UGACGUUCCUUGUAGGAAACU	Cleavage
**bdi-miR398b**	***Bra029784***	75–96	CAGGAGUGUCACUGAGAACACA	**: ::: .::::::..::::**	AGGGUUGCUAGUGACGUUCCUU	Cleavage
**osa-miR2095-3p**	***Bra029784***	58–77	CUUCCAUUUAUGAUAAGUAU	**.: ::: ::.:.: ::::.:**	GUCCUUCUCGUGAUUGGAGG	Cleavage
**aly-miR4248a**	***Bra029784***	158–178	ACAUUUUAUUUUUGGCAAUCA	**.:: ::::.:: ::.:::**	CCGUUUCCAAGAAGAAGAUGA	Cleavage
**aly-miR4248b**	***Bra029784***	158–178	ACAUUUUAUUUUUGGCAAUCA	**.:: ::::.:: ::.:::**	CCGUUUCCAAGAAGAAGAUGA	Cleavage
**aly-miR4248c**	***Bra029784***	158–178	ACAUUUUAUUUUUGGCAAUCA	**.:: ::::.:: ::.:::**	CCGUUUCCAAGAAGAAGAUGA	Cleavage
**gma-miR4363**	***Bra029784***	52–73	CGAUUACCAGAAGGCUUAUUAG	**::.:: . :::::: ::.::.:**	CUGAUCGUCCUUCUCGUGAUUG	Cleavage
**bdi-miR7757-3p.1**	***Bra029784***	192–212	GGUAGUUGAAUGUUUUGUUUA	**::.:::..:.::::: : ::**	GAAGCAAGGCGUUCAAGUUCC	Cleavage
**aly-miR838-3p**	***Bra034084***	152–172	UUUUCUUCUUCUUCUUGCACA	**: : ::::::::::: :::.:**	UUUCCAAGAAGAAGAUGAAGA	Cleavage
**zma-miR399e-5p**	***Bra034084***	130–150	GGGCUUCUCUUUCUUGGCAGG	**::: : :.:::. ::::::::**	CCUCCGAGGAAGAAGAAGCCC	Cleavage
**ath-miR838**	***Bra034084***	152–172	UUUUCUUCUACUUCUUGCACA	**: : ::::::: ::: :::.:**	UUUCCAAGAAGAAGAUGAAGA	Translation
**mtr-miR2673a**	***Bra034084***	159–180	CCUCUUCCUCUUCCUCUUCCAC	**::::: ::::: :.::: :**	GAAGAAGAUGAAGAAGGAGAAG	Cleavage
**mtr-miR2673b**	***Bra034084***	159–180	CCUCUUCCUCUUCCUCUUCCAC	**::::: ::::: :.::: :**	GAAGAAGAUGAAGAAGGAGAAG	Cleavage
**gma-miR4363**	***Bra034084***	43–64	CGAUUACCAGAAGGCUUAUUAG	**::.:: . :::::: ::.::::**	CUGAUCGUCCUUCUUGUGAUCG	Cleavage
**bdi-miR398b**	***Bra034084***	66–87	CAGGAGUGUCACUGAGAACACA	**: ::: .::::::..::::**	AGGGUUGCUAGUGACGUUCCUU	Cleavage
**osa-miR2055**	***Bra034084***	144–163	UUUCCUUGGGAAGGUGGUUUC	**:::.:: ::::.::::: :.:**	GAAGCC-CCUUUCCAAGAAGA	Cleavage
**osa-miR3982-3p**	***Bra034084***	77–97	AGUUGCCUACAUGGAGCGCCA	**:: ::.::: :::.:: ::::**	UGACGUUCCUUGUGGGAAACU	Cleavage
**cca-miR6116-3p**	***Bra034084***	49–69	UCAUUUGAUCACAAGCAUGAG	**:: : :::::::::..: :.**	GUCCUUCUUGUGAUCGGAGGG	Cleavage
**stu-miR8050-3p**	***Bra034084***	182–202	UGACUUGAGAUUCCUACUUGG	**::: :::.: .::::::.**	UGAAGAAGGGAGUUCAAGUUC	Translation
**gma-miR9752**	***Bra034084***	162–182	UGCUUCUUCUUUUCCCUGUUU	**.:: : : :.:::.::::::**	GAAGAUGAAGAAGGAGAAGCU	Cleavage

## Data Availability

Not applicable.

## Supplementary Materials

The following supporting information can be downloaded at: https://www.mdpi.com/article/10.3390/antiox11091782/s1, Table S1: Chinese cabbage S1fa family genes cis-elements.

## References

[1] Mabuchi R., Tanaka M., Nakanishi C., Takatani N., Tanimoto S. (2019). Analysis of Primary Metabolites in Cabbage (*Brassica oleracea* var. capitata) Varieties Correlated with Antioxidant Activity and Taste Attributes by Metabolic Profiling. Molecules.

[2] Gu M., Li N., Ty S., Xh L., Brestic M., Shao H., Li J., Rki S. (2016). Accumulation capacity of ions in cabbage (*Brassica oleracea* L.) supplied with sea water. Plant Soil Environ..

[3] Park S., Valan Arasu M., Lee M.K., Chun J.H., Seo J.M., Lee S.W., Al-Dhabi N.A., Kim S.J. (2014). Quantification of glucosinolates, anthocyanins, free amino acids, and vitamin C in inbred lines of cabbage (*Brassica oleracea* L.). Food Chem..

[4] Yang L., Wu Y., Wang X., Lv J., Tang Z., Hu L., Luo S., Wang R., Ali B., Yu J. (2022). Physiological Mechanism of Exogenous 5-Aminolevulinic Acid Improved the Tolerance of Chinese Cabbage (*Brassica pekinensis* L.) to Cadmium Stress. Front. Plant Sci..

[5] Wang A., Hu J., Gao C., Chen G., Wang B., Lin C., Song L., Ding Y., Zhou G. (2019). Genome-wide analysis of long non-coding RNAs unveils the regulatory roles in the heat tolerance of Chinese cabbage (*Brassica rapa* ssp. chinensis). Sci. Rep..

[6] Anwar A., Kim J.K. (2020). Transgenic Breeding Approaches for Improving Abiotic Stress Tolerance: Recent Progress and Future Perspectives. Int. J. Mol. Sci..

[7] Anwar A., Liu Y., Dong R., Bai L., Yu X., Li Y. (2018). The physiological and molecular mechanism of brassinosteroid in response to stress: A review. Biol. Res..

[8] Gill S.S., Tuteja N. (2010). Reactive oxygen species and antioxidant machinery in abiotic stress tolerance in crop plants. Plant Physiol. Biochem.

[9] Jiang J., Ma S., Ye N., Jiang M., Cao J., Zhang J. (2017). WRKY transcription factors in plant responses to stresses. J. Integr. Plant Biol..

[10] Yang Y., Guo Y. (2018). Unraveling salt stress signaling in plants. J. Integr. Plant Biol..

[11] Sanz A.B., García R., Rodríguez-Peña J.M., Arroyo J. (2017). The CWI Pathway: Regulation of the Transcriptional Adaptive Response to Cell Wall Stress in Yeast. J. Fungi.

[12] Lippold F., Sanchez D.H., Musialak M., Schlereth A., Scheible W.R., Hincha D.K., Udvardi M.K. (2009). *AtMyb41* regulates transcriptional and metabolic responses to osmotic stress in Arabidopsis. Plant Physiol..

[13] Schmidt R., Schippers J.H., Mieulet D., Obata T., Fernie A.R., Guiderdoni E., Mueller-Roeber B. (2013). MULTIPASS, a rice R2R3-type MYB transcription factor, regulates adaptive growth by integrating multiple hormonal pathways. Plant J..

[14] Zagorchev L., Kamenova P., Odjakova M. (2014). The Role of Plant Cell Wall Proteins in Response to Salt Stress. Sci. World J..

[15] Ambawat S., Sharma P., Yadav N.R., Yadav R.C. (2013). MYB transcription factor genes as regulators for plant responses: An overview. Physiol. Mol. Biol. Plants.

[16] Nuruzzaman M., Sharoni A.M., Kikuchi S. (2013). Roles of NAC transcription factors in the regulation of biotic and abiotic stress responses in plants. Front. Microbiol..

[17] Zhao H., Niu Y., Dong H., Jia Y., Wang Y. (2021). Characterization of the Function of Two S1Fa-Like Family Genes From *Populus trichocarpa*. Front. Plant Sci..

[18] Kim S.-I., Lee K.H., Kwak J.S., Kwon D.H., Song J.T., Seo H.S. (2021). Overexpression of Rice *OsS1Fa1* Gene Confers Drought Tolerance in Arabidopsis. Plants.

[19] Zhou D.X., Li Y.F., Rocipon M., Mache R. (1992). Sequence-specific interaction between S1F, a spinach nuclear factor, and a negative cis-element conserved in plastid-related genes. J. Biol. Chem..

[20] Van Zelm E., Zhang Y., Testerink C. (2020). Salt Tolerance Mechanisms of Plants. Annu. Rev. Plant Biol..

[21] Choudhury F.K., Rivero R.M., Blumwald E., Mittler R. (2017). Reactive oxygen species, abiotic stress and stress combination. Plant J..

[22] Wang C., Deng P., Chen L., Wang X., Ma H., Hu W., Yao N., Feng Y., Chai R., Yang G. (2013). A wheat WRKY transcription factor *TaWRKY10* confers tolerance to multiple abiotic stresses in transgenic tobacco. PLoS ONE.

[23] Bo W., Zhaohui Z., Huanhuan Z., Xia W., Binglin L., Lijia Y., Xiangyan H., Deshui Y., Xuelian Z., Chunguo W. (2019). Targeted Mutagenesis of NAC Transcription Factor Gene, *OsNAC041*, Leading to Salt Sensitivity in Rice. Rice Sci..

[24] Ohnishi T., Sugahara S., Yamada T., Kikuchi K., Yoshiba Y., Hirano H.Y., Tsutsumi N. (2005). *OsNAC6*, a member of the NAC gene family, is induced by various stresses in rice. Genes Genet. Syst..

[25] Wei Z.-Z., Hu K.-D., Zhao D.-L., Tang J., Huang Z.-Q., Jin P., Li Y.-H., Han Z., Hu L.-Y., Yao G.-F. (2020). *MYB44* competitively inhibits the formation of the *MYB340-bHLH2-NAC56* complex to regulate anthocyanin biosynthesis in purple-fleshed sweet potato. BMC Plant Biol..

[26] Quan X., Liu J., Zhang N., Xie C., Li H., Xia X., He W., Qin Y. (2021). Genome-Wide Association Study Uncover the Genetic Architecture of Salt Tolerance-Related Traits in Common Wheat (*Triticum aestivum* L.). Front. Genet..

[27] Wang Y., Zhang Y., Zhou R., Dossa K., Yu J., Li D., Liu A., Mmadi M.A., Zhang X., You J. (2018). Identification and characterization of the bZIP transcription factor family and its expression in response to abiotic stresses in sesame. PLoS ONE.

[28] Liu H., Wang Y.X., Li H., Teng R.M., Wang Y., Zhuang J. (2019). Genome-Wide Identification and Expression Analysis of Calcineurin B-Like Protein and Calcineurin B-Like Protein-Interacting Protein Kinase Family Genes in Tea Plant. DNA Cell Biol..

[29] Li S., Miao L., Huang B., Gao L., He C., Yan Y., Wang J., Yu X., Li Y. (2019). Genome-Wide Identification and Characterization of Cucumber BPC Transcription Factors and Their Responses to Abiotic Stresses and Exogenous Phytohormones. Int J Mol Sci.

[30] Liu Q.-L., Xu K.-D., Pan Y.-Z., Jiang B.-B., Liu G.-L., Jia Y., Zhang H.-Q. (2014). Functional Analysis of a Novel Chrysanthemum WRKY Transcription Factor Gene Involved in Salt Tolerance. Plant Mol. Biol. Rep..

[31] Marand A.P., Schmitz R.J. (2022). Single-cell analysis of cis-regulatory elements. Curr. Opin. Plant Biol..

[32] Davoudi M., Chen J., Lou Q. (2022). Genome-Wide Identification and Expression Analysis of *Heat Shock Protein 70* (*HSP70*) Gene Family in Pumpkin (*Cucurbita moschata*) Rootstock under Drought Stress Suggested the Potential Role of these Chaperones in Stress Tolerance. Int. J. Mol. Sci..

[33] Schmitz R.J., Grotewold E., Stam M. (2022). Cis-regulatory sequences in plants: Their importance, discovery, and future challenges. Plant Cell.

[34] Miao Y., Chen K., Deng J., Zhang L., Wang W., Kong J., Klosterman S.J., Zhang X., Aierxi A., Zhu L. (2022). *miR398b* negatively regulates cotton immune responses to *Verticillium dahliae* via multiple targets. Crop J..

[35] Cui C., Wang J.J., Zhao J.H., Fang Y.Y., He X.F., Guo H.S., Duan C.G. (2020). A Brassica miRNA Regulates Plant Growth and Immunity through Distinct Modes of Action. Mol. Plant.

[36] Collin A., Daszkowska-Golec A., Szarejko I. (2021). Updates on the Role of *ABSCISIC ACID INSENSITIVE 5* (*ABI5*) and ABSCISIC ACID-RESPONSIVE ELEMENT BINDING FACTORs (ABFs) in ABA Signaling in Different Developmental Stages in Plants. Cells.

[37] Nath M., Bhatt D., Jain A., Saxena S.C., Saifi S.K., Yadav S., Negi M., Prasad R., Tuteja N. (2019). Salt stress triggers augmented levels of Na^+^, Ca_2_^+^ and ROS and alter stress-responsive gene expression in roots of *CBL9* and *CIPK23* knockout mutants of *Arabidopsis thaliana*. Environ. Exp. Bot..

[38] Zhang H.-f., Liu S.-y., Ma J.-h., Wang X.-k., Haq S.U., Meng Y.-c., Zhang Y.-m., Chen R.-g. (2020). *CaDHN4*, a Salt and Cold Stress-Responsive Dehydrin Gene from Pepper Decreases Abscisic Acid Sensitivity in Arabidopsis. Int. J. Mol. Sci..

[39] Gerik K.J., Donlin M.J., Soto C.E., Banks A.M., Banks I.R., Maligie M.A., Selitrennikoff C.P., Lodge J.K. (2005). Cell wall integrity is dependent on the PKC1 signal transduction pathway in *Cryptococcus neoformans*. Mol. Microbiol..

[40] He Y., Chen Y., Song W., Zhu L., Dong Z., Ow D.W. (2017). A Pap1–Oxs1 signaling pathway for disulfide stress in Schizosaccharomyces pombe. Nucleic Acids Res..

[41] Techo T., Charoenpuntaweesin S., Auesukaree C. (2020). Involvement of the Cell Wall Integrity Pathway of Saccharomyces cerevisiae in Protection against Cadmium and Arsenate Stresses. Appl. Environ. Microbiol..

[42] Zhang Y., Li J., Zhou D., Song J., Gao J. (2022). Nitrogen Uptake and Distribution in Different Chinese Cabbage Genotypes under Low Nitrogen Stress. Int. J. Mol. Sci..

[43] Ying S., Zhang D.F., Fu J., Shi Y.S., Song Y.C., Wang T.Y., Li Y. (2012). Cloning and characterization of a maize bZIP transcription factor, *ZmbZIP72*, confers drought and salt tolerance in transgenic Arabidopsis. Planta.

[44] Ma W., Ren Z., Zhou Y., Zhao J., Zhang F., Feng J., Liu W., Ma X. (2020). Genome-Wide Identification of the *Gossypium hirsutum NHX* Genes Reveals that the Endosomal-Type *GhNHX4A* is Critical for the Salt Tolerance of Cotton. Int. J. Mol. Sci..

[45] Jing Y., Shi L., Li X., Zheng H., Gao J., Wang M., He L., Zhang W. (2019). *OXS2* is Required for Salt Tolerance Mainly through Associating with Salt Inducible Genes, CA1 and Araport11, in Arabidopsis. Sci. Rep..

[46] He L., Jing Y., Shen J., Li X., Liu H., Geng Z., Wang M., Li Y., Chen D., Gao J. (2019). Mitochondrial Pyruvate Carriers Prevent Cadmium Toxicity by Sustaining the TCA Cycle and Glutathione Synthesis. Plant Physiol..

