# Supplementary material for: Genome-Wide Identification and Characterization of Chinese Cabbage S1fa Transcription Factors and Their Roles in Response to Salt Stress
Source: Antioxidants (Basel). 2022 Sep 9;11(9):1782. doi: 10.3390/antiox11091782 (PMC9495863; doi:10.3390/antiox11091782)
Supplement: Supplementary file 1 [file antioxidants-11-01782-s001.zip › Supplementary Table. S1.pdf]

Supplementary Table. S1 Chinese cabbage S1fa family genes cis-elements

Motif name	Function	Bra034084	Bra003132	Bra006994	Bra029784
GATA-motif	Part of a light responsive element	1	0	0	0
LTR	Cis-acting element involved in low-temperature responsiveness	1	1	1	0
TC-rich repeats	Cis-acting element involved in defense and stress responsiveness	1	0	0	2
TCA-element	Cis-acting element involved in salicylic acid responsiveness	1	0	1	1
CGTCA-motif	Cis-acting regulatory element involved in the MeJa-responsiveness	1	2	0	0
ARE	Cis-acting regulatory element essential for the anaerobic induction	3	3	1	1
GT1-motif	Light responsive element	1	0	0	0
TCT-motif	Part of a light responsive element	3	0	2	0
TGACG-motif	Cis-acting regulatory element involved in the meja-responsiveness	1	2	0	0
MBS	MYB binding site involved in drought-inducibility	3	3	0	2
AE-box	Part of a module for light response	2	1	0	1
ABRE	Cis-acting element involved in the abscisic acid responsiveness	0	4	2	2
G-Box	Cis-acting regulatory element involved in light responsiveness	0	6	3	1
TGA-element	Auxin-responsive element	0	2	0	0
CAT-box	Cis-acting regulatory element related to meristem expression	0	1	0	0
TCCC-motif	Part of a light responsive element	0	1	0	0
TCT-motif	Part of a light responsive element	0	1	2	2
LAMP-element	Part of a light responsive element	0	1	0	0
Box II	Part of a light responsive element	0	0	1	1
O2-site	Cis-acting regulatory element involved in zein metabolism regulation	0	0	3	0
CAT-box	Cis-acting regulatory element related to meristem expression	0	0	1	0
TC-rich repeats	Cis-acting element involved in defense and stress responsiveness	0	0	1	0
chs-Unit 1 m1	Part of a light responsive element	0	0	0	1
